# Global research trends of the application of artificial intelligence in bladder cancer since the 21st century: a bibliometric analysis

**DOI:** 10.3389/fonc.2023.1227152

**Published:** 2023-11-29

**Authors:** Yingjian Zhou, Wenchao Xu, Yan Zeng, Hao Li, Zhuo Liu, Tao Wang, Jihong Liu, Hongyang Jiang

**Affiliations:** ^1^ Department of Urology, Tongji Hospital, Tongji Medical College, Huazhong University of Science and Technology, Wuhan, China; ^2^ Institute of Urology, Tongji Hospital, Tongji Medical College, Huazhong University of Science and Technology, Wuhan, China

**Keywords:** bibliometric analysis, bladder cancer, artificial intelligence, robotic surgery, CiteSpace, VOSviewer

## Abstract

**Introduction:**

Since the significant breakthroughs in artificial intelligence (AI) algorithms, the application of AI in bladder cancer has rapidly expanded. AI can be used in all aspects of the bladder cancer field, including diagnosis, treatment and prognosis prediction. Nowadays, these technologies have an excellent medical auxiliary effect and are in explosive development, which has aroused the intense interest of researchers. This study will provide an in-depth analysis using bibliometric analysis to explore the trends in this field.

**Method:**

Documents regarding the application of AI in bladder cancer from 2000 to 2022 were searched and extracted from the Web of Science Core Collection. These publications were analyzed by bibliometric analysis software (CiteSpace, Vosviewer) to visualize the relationship between countries/regions, institutions, journals, authors, references, keywords.

**Results:**

We analyzed a total of 2368 publications. Since 2016, the number of publications in the field of AI in bladder cancer has increased rapidly and reached a breathtaking annual growth rate of 43.98% in 2019. The U.S. has the largest research scale, the highest study level and the most significant financial support. The University of North Carolina is the institution with the highest level of research. EUROPEAN UROLOGY is the most influential journal with an impact factor of 24.267 and a total citation of 11,848. Wiklund P. has the highest number of publications, and Menon M. has the highest number of total citations. We also find hot research topics within the area through references and keywords analysis, which include two main parts: AI models for the diagnosis and prediction of bladder cancer and novel robotic-assisted surgery for bladder cancer radicalization and urinary diversion.

**Conclusion:**

AI application in bladder cancer is widely studied worldwide and has shown an explosive growth trend since the 21st century. AI-based diagnostic and predictive models will be the next protagonists in this field. Meanwhile, the robot-assisted surgery is still a hot topic and it is worth exploring the application of AI in it. The advancement and application of algorithms will be a massive driving force in this field.

## 1 Introduction

Bladder cancer (BCa) is a common urological malignancy of the uroepithelium. As the tenth most common cancer in the world, BCa accounts for over 573,000 new cases and 213,000 deaths annually, with a higher incidence in men compared to women (1). According to the latest cancer statistics from the American Cancer Society, BCa has become the fourth most common malignancy in the American male population (2). Based on the above facts, it is of great significance to achieve early diagnosis and treatment of BCa.

The diagnostic methods for BCa include imaging, cystoscopy, urine tests, etc. and pathological diagnosis is the gold standard (3, 4). BCa can be divided into two types in pathology according to the degree of muscle invasion: non-muscle-invasive BCa and muscle-invasive BCa (5), and prognosis of them is very different. It requires pathologists’ efforts to identify them, but the manual review of pathologist may sometimes bring mistakes (4). Imaging tests can help detect BCa early (6), but they are also influenced by human factors. Treatments for BCa include non-surgical treatments like systemic chemotherapy and radiation therapy and surgical treatments like transurethral resection of bladder tumor, open radical cystectomy, laparoscopic radical cystectomy and robotic-assisted laparoscopic radical cystectomy. Doctors play an important role in treatment selection and prognostic prediction, but this can be influenced by subjective factors (7, 8). Robotic surgery has been widely used in the treatment of BCa, but it is highly dependent on the control of doctors and has not yet been automated. In summary, diagnosis and treatment of BCa can still be improved, and artificial intelligence (AI) brings hope for it.

AI was first proposed in 1956 at Dartmouth Conferences (9) which is the science of research and development for the simulation, extension and expansion of human intelligence. Due to its powerful learning and computational capabilities, AI has already been used in the field of BCa throughout the entire process of medical treatment. In recent years, many AI-based pathological diagnostic models for BCa have been designed (10). A convolutional neural network algorithm-based urinary cytology diagnostic model was shown to be effective in reducing the underdiagnosis rate of low-grade uroepithelial carcinoma (11). The robotic surgery, which has been widely applied in the treatment of BCa in recent years, is also an important area for AI applications. Some studies have reported the use of machine learning to assess surgeons’ robotic surgical skills and stylistic behavior (12, 13). An attention-guided network for surgical instrument segmentation from endoscopic images was invented to improve the accuracy of robotic surgery (14). Meanwhile, the application of AI opens up the possibility of building autonomous surgical robots (15). A study demonstrated a new framework for planning and executing semi-autonomous tissue retraction in robotic surgery based on deep learning and procedural algorithms (16). In addition, AI can also be used to predict the prognosis of treatment, such as recurrence risk and survival prediction (17).

Unfortunately, there are only a few reviews, systematic reviews and meta-analyses that provide a comprehensive analysis of the application of AI in the field of BCa, and only a relatively small number of them have addressed the focus and frontiers. As a visual analysis method, bibliometric analysis has been widely used in different research fields to analyze hotspots (18–26). Its principle is to quantitatively analyze the literature data in a certain research area based on the knowledge of mathematics, statistics, computer science and other disciplines (23, 24). Specifically, bibliometric analysis is used to analyze publications to obtain information about influence in countries/regions, institutions, journals, authors, references, keywords, etc. (25). In addition, it is also used to obtain information about cooperation and relationships by mapping social networks (25). Consequently, to provide an in-depth and reliable analysis of AI application in BCa, bibliometric analysis and visualization of the relevant literature from 2000 to 2022 were performed. We hope this study will help researchers understand the overall framework of the field and today’s research hotspots.

## 2 Methods

### 2.1 Data sources and extraction

Two authors independently searched the literature published between 2000 and 2022 and collected related information on October 21, 2022. It is worth noting that in order to ensure the comprehensiveness of our search, we chose the Web of Science Core Collection (WoSCC), which covers multiple disciplines, rather than PubMed, which focuses mainly on biomedical and health sciences (27). The former has covered some of the most important databases including the Science Citation Index, the Science Citation Index Expanded, the Social Science Citation Index and the Arts and Humanities Citation Index (28). Meanwhile, the strong citation analysis function of WoSCC, which is very suitable for bibliometric analysis, is also one of the important reasons why we chose it (29). A large number of bibliometric analyses published in the past have also confirmed the reliability of our selection (19–22, 24–26).

The following was the search strategy which was finalized after reference of past relevant literature and consultation among all authors: (TS=“bladder cancer” OR “bladder carcinoma” OR “bladder tumour” OR “urothelial cancer of the bladder” OR “urothelial carcinoma of the bladder” OR “urothelial tumour of the bladder” OR “transitional cell cancer of the bladder” OR “transitional cell carcinoma of the bladder” OR “transitional cell tumour of the bladder” OR “Urinary Bladder Neoplasm” OR “Bladder Neoplasm” OR “Urinary Bladder Cancer” OR “Malignant Tumor of Urinary Bladder” OR “Cancer of the Bladder” OR “Cancer of Bladder”) AND (TS=“artificial intelligence” OR “Computational Intelligence” OR “Machine Intelligence” OR “Computer Reasoning” OR “Computer Vision System” OR “Knowledge Acquisition “ OR “Knowledge Representation” OR “Deep Learning” OR “Hierarchical Learning” OR “Machine Learning” OR “Transfer Learning” OR “robotic*” OR “expert* system” OR “intelligent learning” OR “feature* extraction” OR “feature* mining” OR “feature* learning” OR “machine learning” OR “feature* selection” OR “unsupervised clustering” OR “image* segmentation” OR “supervised learning” OR “semantic segmentation” OR “deep network*” OR “Bayes* network” OR “neural network*” OR “neural learning” OR “neural nets model” OR “artificial neural network” OR “data mining” OR “graph mining” OR “data clustering” OR “big data” OR “knowledge graph”) (21, 22).

**Figure 1:**
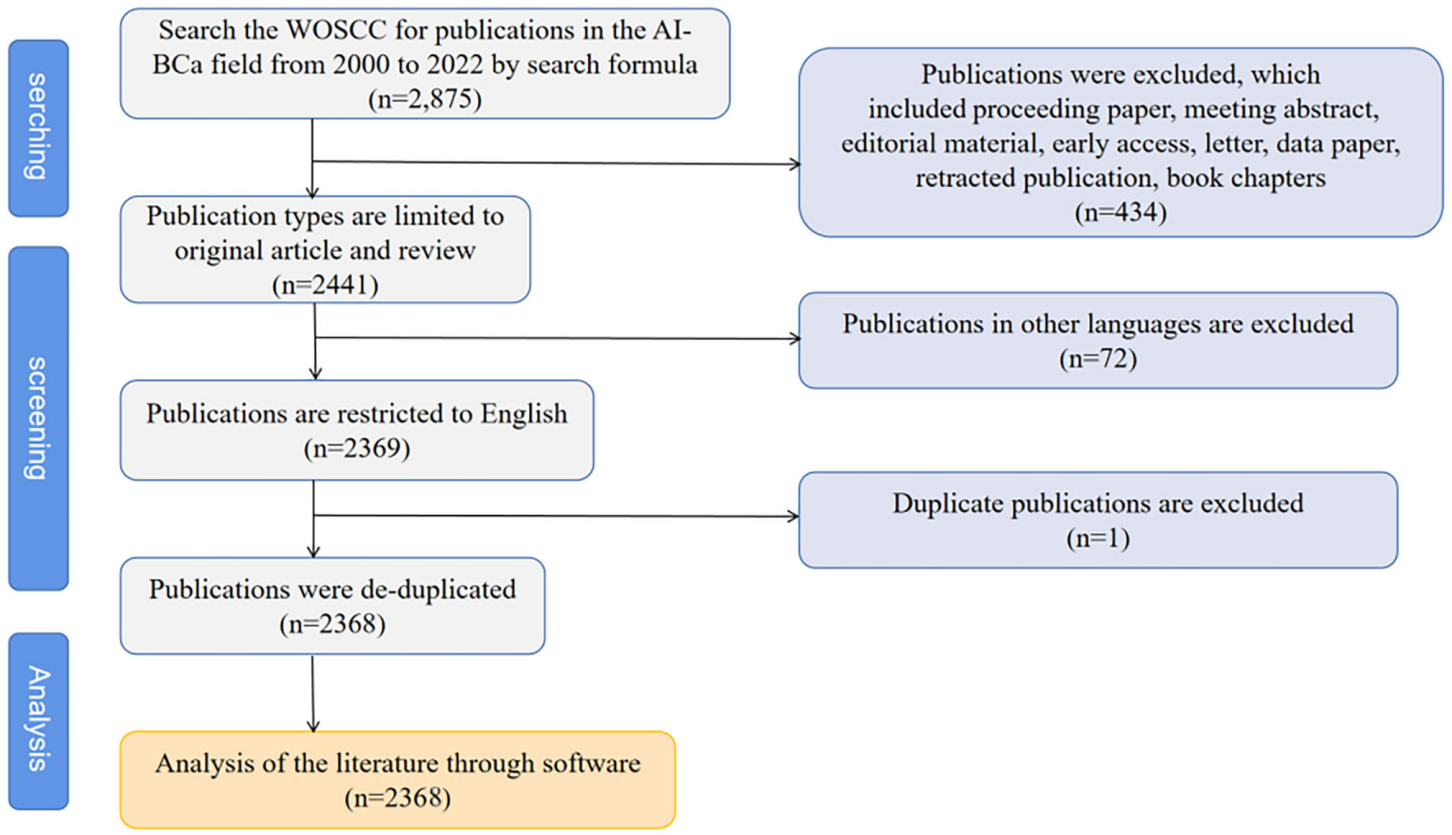
The screening flow chart of this study.

**Figure 1** shows how the search results were made to be more precise. Only documents in the type of articles and reviews and in English were selected. After the authors saved the plain text files of the retrieved documents from WoSCC, CiteSpace V (version 6.1.R2, Drexel University, United States) was used for file de-duplication.

### 2.2 Data analysis and visualization

For bibliometric analysis and visualization, this study mainly used VOSviewer (version 1.6.18) and CiteSpace V (version 6.1.R2 basic). VOSviewer can construct and display large bibliometric maps in different ways including the label, density, cluster density and scatter view (30). Therefore, the co-authorship between country/region, institutions, authors, journals, citation relationships of references, and co-occurrence of keywords were visualized by VOSviewer. The color of nodes represents the sequence of time and the size of nodes reveals the importance of items (30). CiteSpace can be used to analyze and visualize co-citation networks and can also detect and visualize trends and bursts of research (31, 32). Consequently, timelines, co-citation relationships, dual maps of journals and citation bursts of keywords and references were visualized by CiteSpace. In addition, Tableau (version 10.5), online bibliometric analysis platform (https://bibliometric.com/) and online graphing platform (https://echarts.apache.org/) were also used as visualization tools.

Extra data on publication volume, citation frequency, H-index, etc. were extracted from WoSCC. In addition, we conducted CiteSpace analysis to get the centrality and VOSviewer analysis to get the total link strength (TLS). Then we used Microsoft Excel 2019 (Microsoft, Redmond, Washington, United States) to record and analyze all the collected data of unduplicated publications from several aspects including country/region, institution, journal and author.

## 3 Results

### 3.1 Global publications and citations analysis

**Figure 2:**
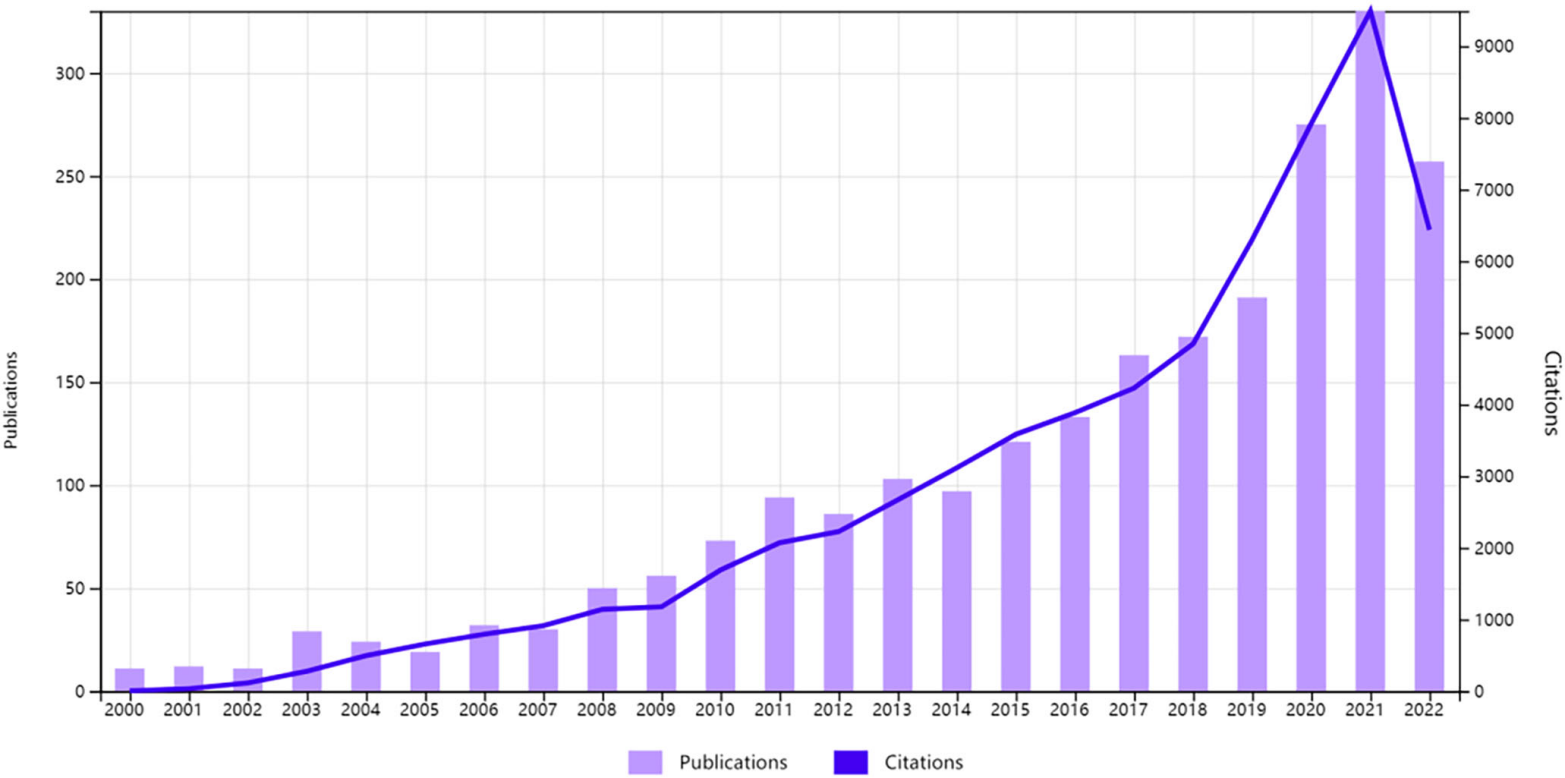
The evolution of the volume and citations of the global publications.

We collected 2368 publications finally and analyzed them in terms of publication volume and citations. According to data on WoSCC, the total citations of AI in the field of BCa was 64,111, and the average citation rate was 27.06 with an H-index of 111 as of October 21, 2022. As shown in **Figure 2**, the number of publications and citations in this field has shown a yearly trend of increase worldwide from 2000 to 2021. The number of publications was only 11 in 2000 but reached 330 in 2021. Amazingly, the annual publication volume increased by 43.98% from 2019 to 2020. According to this trend, the number of publications and citations may reach a new high by the end of 2022.

### 3.2 Countries/regions analysis

**Figure 3:**
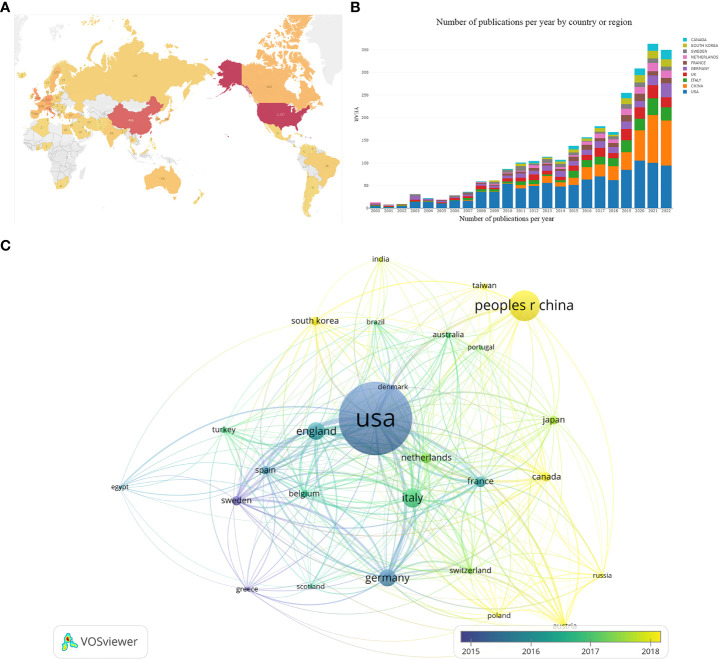
**(A)** A Geo-visualized map built using Tableau based on publication volume by country/region. **(B)** A trend graph based on annual publication volume by country/region. **(C)** An inter-country/regional collaboration map constructed by VOSviewer and based on co-authorship relationships. The thick and thin lines represent the strength of the collaboration. Colors represent the average year of relevant publications in the country/region.

Since the 21st century, a total of 73 countries/regions conducted research on the AI application in the field of BCa. As shown in **Figure 3A**, the countries/regions conducting research in this area are mainly located in North America, East Asia, and Western Europe. China is the only developing country that has conducted extensive research in this field. **Figure 3B** shows the change in annual publication volume for the top 10 country/region in total publications. **Table 1** presents the analysis of publications from these country/region. The United States and China account for the vast majority of these publications. The United States accounted for 43.77% of the total publications worldwide, with 38,126 citations and an H-index of 96. Regarding average citations per paper, Sweden, Germany and UK are in the top three. In terms of the H-index, USA, UK, and Germany are in the top three. USA, UK, and Italy have the highest TLS in the cited relationships.

**Table 1:** Information about the ten most published countries in the field of AI application in BCa.

Rank	Country	Counts	Percentage	H-index	Total citations	Average citation per paper	Centrality	TLS
1	USA	1037	43.77%	96	38,126	36.77	0.13	12969
2	CHINA	456	19.25%	38	5,989	13.13	0.01	2531
3	ITALY	238	10.05%	42	6,633	27.87	0.06	4239
4	UK	230	9.71%	50	10,210	44.39	0.18	5995
5	GERMANY	211	8.91%	48	9,655	45.76	0.13	3895
6	FRANCE	126	5.32%	34	5,510	43.73	0.11	1970
7	NETHERLANDS	115	4.86%	28	4,447	38.67	0.04	1666
8	SWEDEN	107	4.52%	38	5,562	51.98	0.05	4172
9	SOUTH KOREA	104	4.39%	23	2,170	20.87	0.06	1614
10	CANADA	103	4.35%	25	3,629	35.23	0.06	1228

Co-authorship relationships between countries/regions are shown in **Figure 3C**. The United States has worked with Italy, England, Germany, Sweden, China, and Canada on no less than 53 occasions. Among these, the U.S. cooperates most with Italy, reaching 103 times. In addition, there are up to 53 collaborations between Italy and Germany. As shown in **Figure 3C**, the node’s color represents the average year of research conducted by the country/region in the field of AI application in BCa. Sweden, the United States, and Germany were among the first countries to enter the area and achieved a high level of influence. China started research in this field late but has already published more than the U.S. in 2021 and 2022.

### 3.3 Research institutions and funding agencies analysis

**Figure 4:**
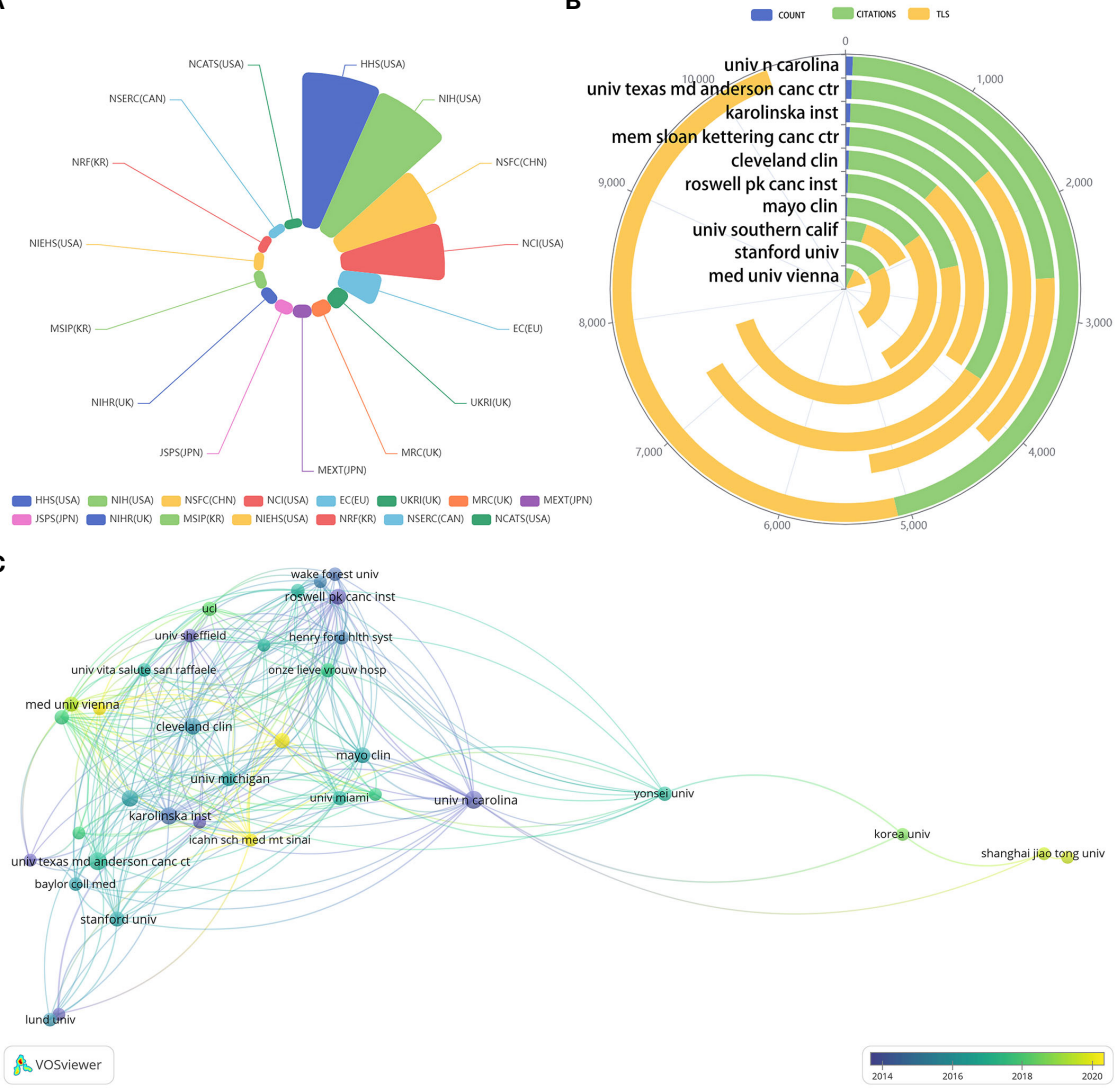
**(A)** The percentage of publications from top 15 funding agencies. **(B)** The publication volume, TLS, and citations of the top 10 research institutions. **(C)** A graph of co-authorship between different research institutions.

**Figure 4A** showcases the top 15 funding organizations in terms of publication volume. These funding agencies are all from high publication volume regions/countries including the US (5), UK (3), Japan (2), Korea (2), EU (1), Canada (1) and China (1). **Figures 4B, C**, show the TLS, total citations and publications of the top 10 research institutions and the collaboration between the research institutions. **Table 2** presents more detailed information about the top 10 research institutions. The total number of research institutions with a publication volume more significant than five articles is 327. Roswell Park Comprehensive Cancer Center (5319), University of North Carolina (5295) and Karolinska Institutet (3710) have the highest TLS. University of North Carolina (5045), Memorial Sloan Kettering Cancer Center (3731) and The University of Texas MD Anderson Cancer Center (2602) have the most citations. According to the node’s color in **Figure 4C**, University of North Carolina, The University of Texas MD Anderson Cancer Center and Roswell Park Comprehensive Cancer Center were the first research institutions to enter the field of AI application in BCa. In addition, with the highest number of publications (56) and the highest average citations per paper (90.09), University of North Carolina is considered to be the most influential research institution.

**Table 2:** Information about the top 10 research institutions.

Institution	Country	Publications volume	Citations	Average citations per paper	TLS
University of North Carolina	USA	56	5045	90.09	5295
The University of Texas MD Anderson Cancer Center	USA	54	2602	48.18	1525
Karolinska Institutet	Sweden	48	1497	31.19	3710
Memorial Sloan Kettering Cancer Center	USA	46	3731	81.10	3527
Cleveland Clinic	USA	43	1222	28.42	2503
Roswell Park Comprehensive Cancer Center	USA	40	2345	58.63	5319
Mayo Clinic	USA	38	1610	42.37	2952
Stanford University	USA	37	1812	48.97	2753
Medical University of Vienna	Austria	37	733	19.81	1471
University of Southern California	USA	37	526	14.22	1345

### 3.4 Journals analysis

**Figure 5:**
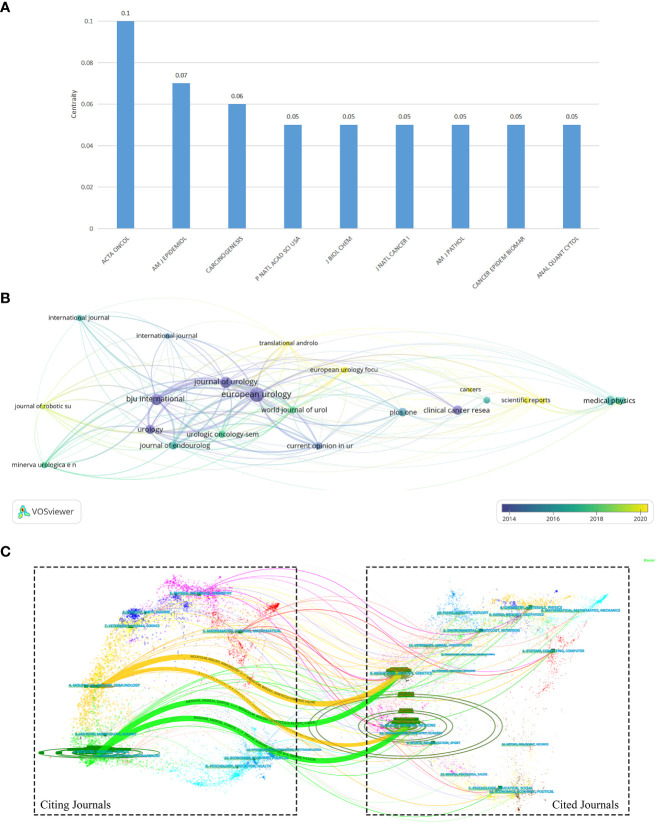
**(A)** The nine journals with betweenness centrality above 0.05 in the co-citation analysis. **(B)** Visualization of the citation relationships between journals by VOSviewer. **(C)** A journal dual-map produced by CiteSpace, showing the citation and cited relationships between journals.

**Figure 5A** shows journals with betweenness centrality above 0.05 but only one with a centrality of 0.1. ACTA ONCOLOGICA has the highest centrality (0.1), while AMERICAN JOURNAL OF HUMAN GENETICS (0.07) and CARCINOGENESIS (0.06) are in the second and third place. **Table 3** lists the top 10 most published journals in the field. The top three are EUROPEAN UROLOGY, BJU INTERNATIONAL, and JOURNAL OF UROLOGY. With an impact factor of 24.267 and a total citation of 11,848, EUROPEAN UROLOGY is the most influential journal in the field of AI application in BCa and has achieved an H-index of 62. **Figure 5B** shows a graph of journal citation relationships produced by VOSviewer. EUROPEAN UROLOGY, BJU INTERNATIONAL, and JOURNAL OF UROLOGY have the strongest TLS with 2641, 1397, and 1026, respectively. And **Figure 5C** shows the citation and cited relationships between journals. It can be seen that publications in journals in the fields of Molecular/Biology/Immunology and Medical/Medicine/Clinical often cite publications in journals in the areas of Molecular/Biology/Immunology and Health/Nursing/Medicine.

**Table 3:** The top 10 most published journals in the field of AI application in BCa.

JOURNAL	Country	Publications	IF	JCR	H-index	Total citations	TLS	Average citations per paper
EUROPEAN UROLOGY	NETHERLANDS	94	24.267	Q1	62	11848	2641	126.04
BJU INTERNATIONAL	UK	90	5.969	Q1	39	3887	1397	43.19
JOURNAL OF UROLOGY	USA	76	7.600	Q1	35	3657	1026	48.12
JOURNAL OF ENDOUROLOGY	USA	61	2.619	Q3	19	946	669	15.50
UROLOGY	USA	57	2.633	Q3	25	1629	634	28.58
WORLD JOURNAL OF UROLOGY	USA	53	3.661	Q2	16	593	468	11.19
MEDICAL PHYSICS	USA	50	4.506	Q2	20	1478	215	29.56
UROLOGIC ONCOLOGY SEMINARS AND ORIGINAL INVESTIGATIONS	USA	49	2.954	Q3	13	553	401	11.28
CURRENT OPINION IN UROLOGY	UK	44	2.808	Q3	13	487	434	11.07
FRONTIERS IN ONCOLOGY	Switzerland	43	5.738	Q2	10	337	108	7.84

### 3.5 Authors analysis

**Figure 6:**
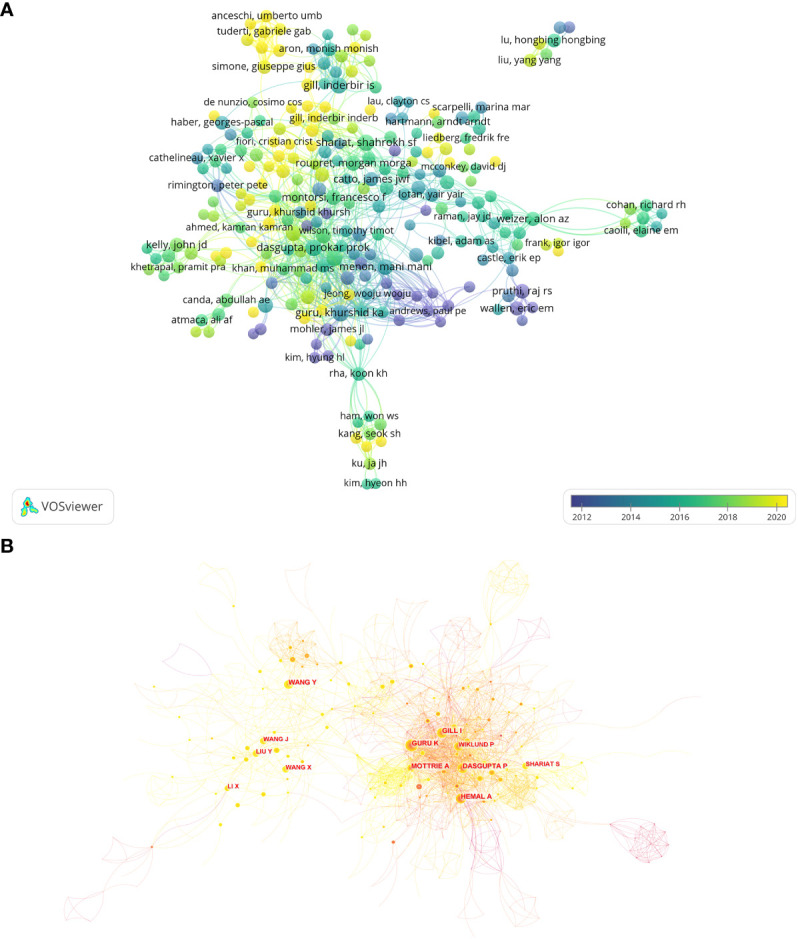
**(A)** Visualization of co-authorship between authors by VOSviewer. **(B)** The author’s co-citation analysis graph by CiteSpace.

A total of 13,036 researchers have been involved in publishing papers about AI application in the field of BCa. A total of 38 of these researchers have published no less than 15 ones. **Table 4** presents the top 10 researchers with the highest volume of publications. They come from the USA (6), UK (2), Sweden (1), and Austria (1) and are the most active authors in this field. In Addition, these authors are from high-level research institutions. In terms of average citations per paper, Menon, Pruthi, and Peabody have an average of over 80 citations per paper. Wiklund from Karolinska Institutet has the highest number of publications and TLS. Menon has the highest number of total citations. Guru has the highest H-index, which means he is highly influential in this field. **Figure 6A** demonstrates the collaboration between authors. Wiklund and Menon have extensive partnerships with researchers in several different clusters. **Figure 6B** shows the co-citation relationships between authors. The CiteSpace analysis showed that Guru and Rha had the highest centrality at 0.09. In addition, MOTTRIE is also an essential node with high centrality (0.08).

**Table 4:** The top 10 authors with the highest publication volume in the field of AI application in BCa.

Rank	Author	Country	Institution	Publications	Total citations	Average citation per paper	H-idex	TLS
1	Wiklund, P.	SWEDEN	Karolinska Institutet	52	2,677	51.48	26	564
2	Guru, Khurshid A.	USA	Roswell Park Comprehens Canc Ctr	46	2,415	52.5	27	531
3	Hemal, Ashok K.	USA	Wake Forest University	41	2,018	49.22	23	141
4	Dasgupta, Prokar	UK	Guy's & St Thomas' NHS Foundation Trust	40	2,054	51.35	26	469
5	Gill, Inderbir	USA	Keck Sch Med	38	1,385	36.45	22	210
6	Shariat, Shahrokh F.	AUSTRIA	Medical University of Vienna	35	931	26.6	16	440
7	Menon, Mani	USA	Henry Ford Hospital	34	2,885	84.85	25	410
8	Pruthi, Raj S.	USA	Mayo Clinic	29	2,382	82.14	20	210
9	Peabody, James O.	USA	Henry Ford Hospital	27	2,365	87.59	20	263
10	Catto, James W. F.	UK	University of Sheffield	26	2,080	80	19	184

### 3.6 References analysis

**Figure 7:**
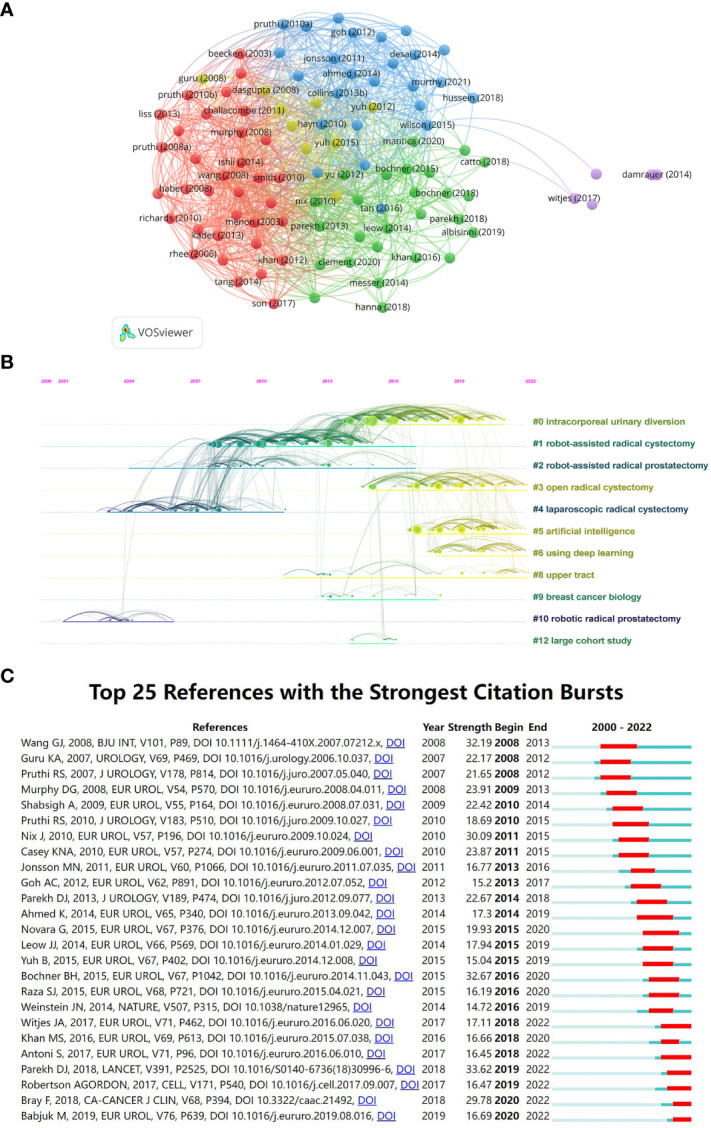
**(A)** Visualization of citation relationships between references by VOSviewer. **(B)** A timeline of research hotspots in the field of AI application in BCa. **(C)** The top 25 references with the strongest citation bursts.

This study was based on 2368 references (article and review) for bibliometric analysis. A total of 134 papers were cited more than 100 times. We combined the results of the bibliometric analysis, read the titles and abstracts of the articles, and discussed among all authors to finally compile and list the top 10 most influential original studies in the field of AI application in BCa (**Table 5**). These studies come from different countries including the U.S (7), China (1), France (1) and Denmark (1). Menon (2003), Nix (2010), and Dyrskjot (2003) have the highest number of citations, with 402, 387, and 386, respectively (33–35). We visualized the references’ citation relationships by Vosviewer and the co-citation relationships by CiteSpace (**Figures 7A**; **S1**). As shown in **Figure 7A**, Nix (2010), Novara (2015), and Menon (2003a) have the highest Links, with 177, 161, and 160, respectively (33, 34, 36). Through analysis, Zhang (2017), Challacombe (2011), and Bruins (2014) have the highest centrality with 0.17, 0.15, 0.15, respectively, and are significant nodes in the co-citation relationship (37–39).

**Table 5:** The top 10 most influential original articles in the field of AI application in BCa.

Title	Journals	Country	Fisrt author	Year	Citations
Nerve-sparing robot-assisted radical cystoprostatectomy and urinary diversion	BJU INTERNATIONAL	USA	Menon, M	2003	402
Prospective Randomized Controlled Trial of Robotic versus Open Radical Cystectomy for Bladder Cancer: Perioperative and Pathologic Results	EUROPEAN UROLOGY	USA	Nix, Jeff	2010	387
Identifying distinct classes of bladder carcinoma using microarrays	NATRUE GENETICS	Denmark	Dyrskjot, L	2003	386
A Consensus Molecular Classification of Muscle-invasive Bladder Cancer	EUROPEAN UROLOGY	France	Kamoun, Aurelie	2020	381
Comparing Open Radical Cystectomy and Robot-assisted Laparoscopic Radical Cystectomy: A Randomized Clinical Trial	EUROPEAN UROLOGY	USA	Bochner, Bernard H.	2015	362
Robot-assisted radical cystectomy versus open radical cystectomy in patients with bladder cancer (RAZOR): an open-label, randomised, phase 3, non-inferiority trial	LANCET	USA	Parekh, Dipen J.	2018	361
Bladder cancer outcome and subtype classification by gene expression	CLINICAL CANCER RESEARCH	USA	Blaveri, E	2005	256
A Radiomics Nomogram for the Preoperative Prediction of Lymph Node Metastasis in Bladder Cancer	CLINICAL CANCER RESEARCH	China	Wu, Shaoxu	2017	227
Robotic vs openradical cystectomy: prospective comparison of perioperative outcomes and pathological measures of early oncological efficacy	BJU INTERNATIONAL	USA	Wang, Gerald J.	2008	224
A Comparison of Postoperative Complications in Open versus Robotic Cystectomy	EUROPEAN UROLOGY	USA	Casey, K. Ng	2010	200

**Figure 7B** visualizes a timeline of research hotspots in the field of AI application in BCa. By the calculation of CiteSpace, the Modularity Q is equal to 0.788, and Silhouette S is equal to 0.9411, which indicates that the clustering structure is significant and reasonable. In recent years, intracorporeal urinary diversion, open radical cystectomy, artificial intelligence and using deep learning have been scorching research topics in this field. **Figure 7C** lists the top 25 references with the strongest citation bursts. Most of the references with the citation bursts were related to robotic-assisted surgery. The first reference with citation bursts appeared in 2008 and was associated with the clinical evaluation of open radical cystectomy versus robotic-assisted radical cystectomy (40).

### 3.7 Keywords analysis

**Figure 8:**
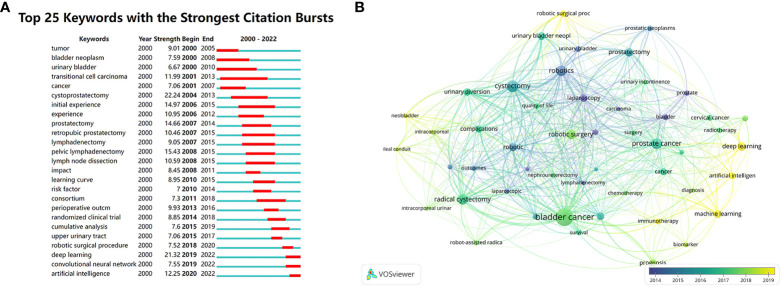
**(A)** The top 25 keywords with the strongest citation bursts. **(B)** Visualization of the co-occurrence analysis of keywords by VOSviewer.

A total of 6611 author keywords were included in this study. **Figure 8A** shows the top 25 keywords with the strongest citation bursts. The citation bursts for Tumor, Bladder neoplasm and Urinary bladder indicate that the literature we searched fits our research on BCa. Deep learning, convolutional neural network and artificial intelligence have gotten citation bursts in recent years. Research related to AI algorithms for BCa started to burst into growth in 2019, which was related to the rapid development of AI algorithm in recent years. As shown in **Figure 8B**, nodes of deep learning, artificial intelligence, machine learning, robotic surgery, etc. are colored in yellow, indicating that they are the most recent keywords. Among them, the robotic surgery takes the center stage. Besides, we list the top 10 most frequently occurring keywords (**Figure S2**). These keywords are all related to surgery or AI algorithms. The emergence of prostate cancer is due to the early use and high level of AI in prostate cancer-related fields, with the da Vinci robot being used in radical prostatectomy (41). In addition, this is also related to the proximity between the bladder and the prostate, with procedures often involving the both. Because they are both in the treatment area of urology, the bladder and the prostate are often studied simultaneously.

## 4 Discussion

Researchers need to update their professional knowledge by reading a large number of documents. Bibliometric analysis helps to clearly show the research progress and hotspots in relevant fields.

Breakthroughs in machine learning algorithms were made in 2013, leading to the rapid expansion of AI into almost all fields. By 2016, AI had begun to cover many medical areas (42). Supportive policies from governments and funding agencies have also provided funding for the growth in the field of AI application in BCa (42). In 2019, the global publication volume in this field experienced a surge, with an impressive growth rate of 43.98%. This field continues to rapidly increase and is considered a research area with great potential.

In terms of countries/regions, the country/region involved in research about AI application in BCa are mainly concentrated in North America, East Asia, and Europe. These regions are the most economically and technologically advanced regions in the world. But the development level and scale of research about AI application in BCa in developing countries are not high, which indicates that research needs sufficient funding and mature science and technology to support it. In addition, China is the only developing country in the top 10. Notably, China surpassed the US in the annual publishing volume in 2021 and 2022, and has maintained a high rate of increase. It can be expected that China will invest more money and effort in this field and may achieve more influence in the future.

Regarding country/region cooperation, cooperation between countries/regions occurs mainly between developed countries in North America and Europe. Specifically, the U.S. is at the center of global collaboration, with significant cooperation with China, Italy, Canada, the UK, and Germany. In addition, Germany, Italy, and the UK also have very high TLS in co-authorship analysis, indicating their essential role in global collaboration. This collaboration facilitates the output of high-level research and the explosion of progress in cutting-edge science and technology. However, it can also lead to a high scientific and technological disparity between countries, resulting in more severe scientific and technical polarization. To solve this, developing countries should proactively seek international cooperation.

Of the top 10 research institutions, eight are from the United States, one is from Austria, and one is from Sweden. With the highest number of publications (56), the highest citation rate (5045), and the highest per-page citation rate (90.09), the University of North Carolina is considered the most influential research institution in the field of AI application in BCa. The University of North Carolina has been working in this field since 1999, focusing on robotic-assisted radical cystectomy, robotic-assisted urinary diversion surgery, BCa prediction models, and AI algorithms for radionics. Roswell Park Comprehensive Cancer Center has the highest TLS (5319). Although Roswell Park Comprehensive Cancer Center entered this field later (2007), it achieved high-quality research (average citations per paper = 58.63) with a research focus similar to the University of North Carolina. Two European institutions are in the top 10 worldwide in terms of publication volume. The average citations per paper were 19.81 for Medical University of Vienna and 31.19 for Karolinska Institutet, which still have a gap with the top institutions in the U.S. Currently, there are also many top funding institutions in East Asia, including three from Japan and one each from Korea and China. This suggests that East Asia will have a solid competitive edge in the field of AI application in BCa in the future.

The bibliometric analysis of journals is essential. Through this section, researchers can learn about the journals gathering high level research about AI application in BCa and get access to research hotspots. In addition, this will be a guidance for researchers to submit their manuscripts. **Table 3** summarizes the top 10 most published journals. These journals focus on the field of urology as well as oncology. EUROPEAN UROLOGY, BJU INTERNATIONAL and JOURNAL OF UROLOGY are the most critical journals in genitourinary systems as Q1 journals in JCR division. It is clear from **Figure 5B** that four journals, including EUROPEAN UROLOGY, BJU INTERNATIONAL, JOURNAL OF UROLOGY, and UROLOGY, are at the center of the citation relationship. Among them, EUROPEAN UROLOGY is, deservedly, an excellent journal for researchers in the field of AI application in BCa to study, which has the highest number of publications in this field with 11,848 citations. Although BJU INTERNATIONAL, JOURNAL OF UROLOGY, and UROLOGY did not achieve exceptionally high impact factors in 2021, they all had an average citation rate per paper of more than 28. Therefore, these journals have a strong influence within this field and are equally worthy of studying and finding hotspots for researchers.

**Table 4** summarizes the top 10 authors in terms of publication volume. Seven of these researchers are from the United States. Wiklunk from Karolinska Institutet has the highest publication volume and total number of citations in the field of AI application in BCa. Guru, from Roswell Park Comprehensive Cancer Center, has the highest H-index in this field. Although Menon, Pruthi, and Peabody do not have more than 35 publications, they have an average of more than 82 citations per paper and are considered high-impact authors in the field. The four researchers focused their research on the following areas: robotic-assisted radical cystectomy, intracorporeal urinary diversion, quality of life, assisted chemotherapy and predictive modeling. A large portion of these are clinical studies related to robotic-assisted surgery. In addition, Guru has done more research on predictive modeling, urinary diversion and lymphadenectomy and has achieved more significant results (43–47). In recent years he has done much research on Neoadjuvant Chemotherapy (48–50). Menon and Peabody are colleagues at Henry Ford Hospital, and their research focuses on robotic surgery and urinary diversion surgery. This demonstrates the extremely high level of robotic surgery and research experience of Henry Ford Hospital. The three authors with the highest centrality are Guru, Rha, and Mottrie. Rha is from South Korea and has extensive collaborations with high-level researchers in Europe and the United States. For researchers in the field of AI application in BCa, we believe that learning from the work of these high-level researchers and actively participating in collaborative research projects will tremendously affect research capabilities.

Reference analysis is significant in finding highly cited publications and new research hotspots. **Table 5** summarizes the top 10 most highly cited papers related to the field of AI application in BCa. They are mainly from three journals: EUROPEAN UROLOGY, BJU INTERNATIONAL and CLINICAL CANCER RESEARCH. The earliest published highly cited literature was Menon’s 2003 clinical study describing a technique of nerve-sparing robot-assisted radical cystoprostatectomy for patients with BCa (33). In the same year, Dyrskjot published a paper on BCa classification based on supervised algorithms in NATRUE GENETICS (35). These two papers are considered pioneering works in the field of AI application in BCa and have induced two different routes in the area at a later stage which are robotic surgery research and diagnostic and predictive models. Wu (2017), Parekh (2018), and Kamoun (2020) are highly cited documents that have emerged in recent years and are more representative of current research hotspots (51–53). These three papers mainly relate to radiomic prediction, robotic surgery evaluation, and consensus molecular classification. It is worth noting that these research directions are, in fact, a continuation of the two 2003 papers mentioned above. This suggests that research about the robotic surgery and AI diagnostic and predictive models has continued to emerge with new technologies over the past 23 years and is likely to grow explosively in the coming years.

**Figure 7C** summarizes the top 25 documents with the strongest citation bursts. To get more helpful research guidance, we focused more on the literature with a citation burst in 2022. Through burst detection and our screening, we chose to analyze the literature published by Robertson in 2017 and Parekh in 2018 (52, 54). Similar to previous findings, these two papers focus on robotic surgery research and AI diagnostic and predictive models. The study by Robertson et al., published in Cell, identified subgroups with differential epithelial-mesenchymal transition status, *in situ* carcinoma score, histological features, and survival by clustering the expression of mRNA, lncRNA and miRNA (54). And this was achieved based on many AI algorithms, such as the MutSig algorithm, ABSOLUTE algorithm, HLA typing algorithm, PathSeq algorithm, RBN algorithm, etc. And the study of Parekh et al., published in LANCET, evaluated robot-assisted radical cystectomy versus open radical cystectomy by the RAZOR trial (52). These articles still have citations explosion in 2022, indicating that robotic-assisted surgery and AI diagnostic and predictive models for BCa are still hot research topics. To find more research hotspots, we plotted a timeline of the references for the field of AI application in BCa (**Figure 7B**). Since 2013, the intracorporeal urinary diversion and open radical cystectomy have been the most explosive research hotspots on the surgery. Open radical cystectomy is a traditional surgical procedure for treatment of the BCa, but in recent years research on this procedure has still received frequent citations. From reading the relevant literature, we found that this burst is mainly due to the clinical comparison of open cystectomy versus robot-assisted cystectomy in recent years (52, 55, 56). And the robotic-assisted surgery has shown a significantly better postoperative quality of life and less blood loss (55). It can also be seen that artificial intelligence and deep learning-related documents have been widely emerging and cited since 2016. This was related to the rise of AI and the rapid advancement of related algorithms. AI diagnostic and predictive models need algorithmic improvements, which can explain the explosion of research in AI diagnostic models during this period.

We likewise performed a citation burst analysis of keywords (**Figure 8A**). Robotic surgical procedure, deep learning, convolutional neural network and artificial intelligence have had citation bursts in recent years. These are all keywords related to AI programming, which suggests that algorithms are of greater importance for future research about AI application in BCa. In addition, we performed a co-occurrence analysis of the keywords (**Figure 8B**). These keywords provide researchers with direct indication of research hotspots. Future hotspots in the field of AI application in BCa may be toward diagnostic and predictive models for BCa and improving robotic surgical procedures which require more programmers to work harder to achieve powerful algorithmic and higher accuracy. AI diagnostic and predictive models for BCa have been extensively studied in the last few years. Meanwhile, since 2019, convolutional neural networks and deep learning have triggered an intense citation explosion. This confirms that the AI model for pathology diagnosis and prediction are a hot research topic that has emerged in recent years. From 2019 to 2021, a large number of AI algorithm-based models for BCa diagnosis and prediction were developed, which include almost all aspects of BCa diagnosis and prediction: cytological diagnosis, histological diagnosis, prognosis prediction, automated grading, etc. (11, 57–60). Notably, testing of these models showed extremely high AUC and even detected low-grade BCa that pathologists missed. These provide essential guidance for radiation therapy and chemotherapy in managing the BCa. However, these models are not perfect, which means that they deserve to be further improved. According to the literature, the algorithms used in these models are mainly machine learning, deep learning and convolutional neural networks, and machine learning is currently one of the most mentioned techniques in the field of AI (61). Therefore, we recommend that researchers pay more attention to these techniques and improve the old or develop new models based on them. Regarding the diagnosis of BCa, a large number of studies have focused on the use of AI for pathological image analysis, but recently research on AI application in the cystoscopy has begun to emerge (62–64). One of the difficulties of this kind of research is to achieve the real-time detection of images (65), which may become a new research direction. Regarding the prognosis prediction of BCa, research is mainly focused on the prediction of survival, recurrence, treatment response, etc. (17) Among them, the application of deep learning is worth paying attention to, and the use of multimodal learning may become a future research direction (66). At the same time, the robotic surgery is still a high research hotspot today. The robotic surgery has always been a key application area of AI in medicine and thus has attracted the attention of many researchers (12–16, 67, 68). Notably, current research hotspots are turning to robotic-assisted radical cystectomy versus open radical cystectomy and intracorporeal urinary diversion (44, 45, 52, 55). These studies mainly focused on surgical outcomes and prognosis with little direct discussion about AI, but their results suggest that the robotic surgery is still worth improving, and focusing on the application of AI can provide ideas for this. An article focused on the hotspots and trends in the field of AI revealed that robots were a highly concerned topic in this field, including human-robot interaction, robot manipulation, robot grasp design, etc. (61) This suggests that AI has a broad application prospect in surgical robots, and researchers can make new explorations based on the above directions. Published research on AI and surgical robots is also worth referencing, including directions such as autonomous surgical robots, motion analysis, and presurgical planning (15, 67).

In short, the analysis of keywords brings us the following insights: the robot-assisted surgery for BCa radical treatment and urinary diversion is still a focus of the field of AI application in BCa, and the diagnostic and predictive models of AI for BCa can lead the subsequent explosion in this field. It is worth emphasizing that we also need to pay attention to the limitations of research on AI application in BCa, which can help us to be clearer about the future direction of improvement and development. Firstly, some studies of AI diagnostic and predictive models mentioned the need for prospective studies to further verify the reliability of these models (11, 57), suggesting that they cannot be used in current clinical practice. There are some other factors that hinder the integration of AI into clinical settings, such as the lack of generalizability across different datasets (58), the lack of availability and quality of datasets (65), and so on. In addition, information security issues may arise with the rapid development of AI and big data technology, which will further raise ethical issues and affect patient acceptance.

### 4.1 Limitations

Although bibliometric analysis has many attractive advantages, our research still has limitations. Firstly, the inclusion of the literature was not comprehensive. This was due to the fact that we merely included English literature and WoSCC was our only source of data which can’t cover all journals of one discipline. Secondly, the keywords plus of WoSCC might result in the inclusion of the literature on other cancers such as the prostate cancer. Both of these may lead to bias.

## 5 Conclusion

AI application in BCa is currently a trendy research area and research on it has been increasing since 2000. The hot research topics in this area include two main parts: AI models for the diagnosis and prediction of BCa and novel robotic-assisted surgery for BCa radicalization and urinary diversion. We venture to speculate that AI models for the diagnosis and prediction of BCa will be the next great hotspot in urology. In addition, improving and applying algorithms will be a strong driving force for development of this field. To achieve these, significant funding and the combined efforts of programmers and medical practitioners will be urgently needed.

## Data availability statement

Publicly available datasets were analyzed in this study. This data can be found here: https://webofscience.clarivate.cn/wos/

## Author contributions

HJ and YJZ determined the topic and designed the study. WX and YZ searched and referred documents. HL, ZL and JL collected data. YJZ, WX, YZ, and TW analyzed data and mapped by the softwares. YJZ, WX and YZ drafted the manuscript. HJ was responsible for revision and correspondence.
