# Supplementary material for: Global research trends of the application of artificial intelligence in bladder cancer since the 21st century: a bibliometric analysis
Source: Front Oncol. 2023 Nov 29;13:1227152. doi: 10.3389/fonc.2023.1227152 (PMC10718619; doi:10.3389/fonc.2023.1227152)
Supplement: Supplementary file 1 [file DataSheet_1.pdf]

Supplementary Material

1 Supplementary Figures

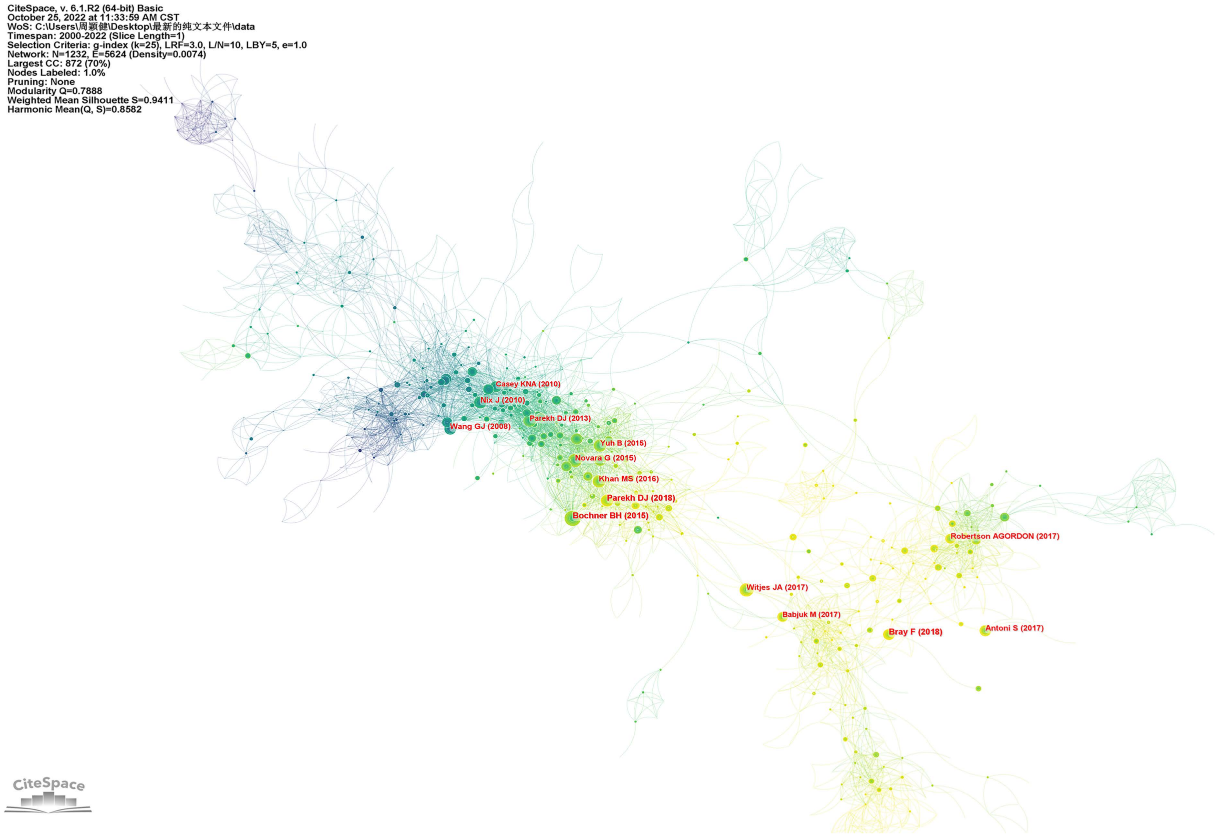

Supplementary Figure S1. The co-citation relationships between references visualized by VOSviewer.

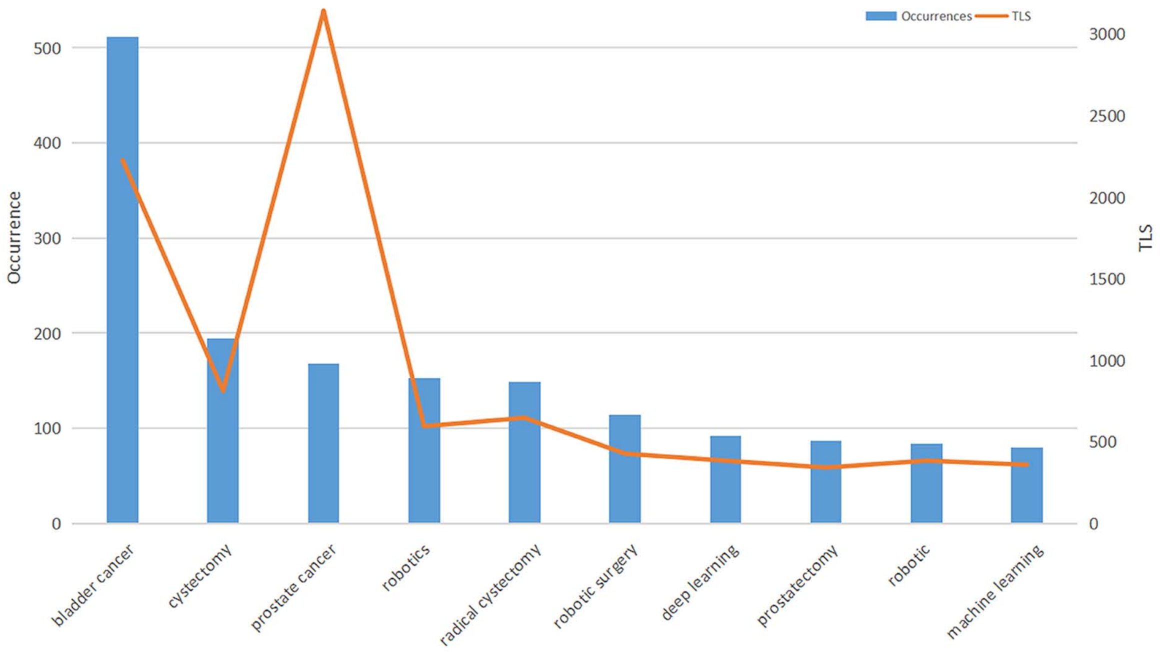

Supplementary Figure S2. The top 10 most frequently occurring keywords.
